# Editorial: Innovations in biologically active nutrients extraction and delivery

**DOI:** 10.3389/fnut.2024.1425911

**Published:** 2024-05-20

**Authors:** Ahmed A. Zaky, Pilar Gómez-Cortés, Blanca Hernández-Ledesma

**Affiliations:** ^1^Department of Food Technology, National Research Centre, Food Industries and Nutrition Research Institute, Cairo, Egypt; ^2^Department of Food Engineering and Process Management, Institute of Food Sciences, Warsaw University of Life Sciences - SGGW, Warsaw, Poland; ^3^Department of Bioactivity and Food Analysis, Institute of Food Science Research (CIAL, CSIC-UAM), Madrid, Spain

**Keywords:** green extraction technologies, by-products, food applications, bioactive, health benefits

Bioactive compounds have been discussed for a while, but it has recently become one of the most popular topics in research and food communities. Among bioactive compounds, proteins, peptides, lipids, carbohydrates, and antioxidant molecules in food have drawn increasing attention due to their beneficial impacts on human health (1). These substances are known to control the progression of chronic diseases through their antioxidant, anticancer, antimicrobial, and anti-inflammatory properties. The development of food products with natural extracts, including polyunsaturated fatty acids, bioactive peptides, or phenolic compounds, can be considered an innovative strategy not only to boost their nutritional quality but also to attract consumers and influence food marketing. Extraction is essential in obtaining bioactive compounds from food sources, including by-products. Thus, the development of environmentally friendly and sustainable extraction techniques is a significant topic in food science and others (1, 2). In this context, the extraction using non-thermal techniques (e.g., ultrasound-assisted extraction, UAE) and thermal techniques (e.g., microwave-assisted extraction, MAE), as well as the incorporation of those compounds to foods are challenging due to the complex composition of the different food matrices.

This Research Topic culminated in a total of 16 submissions dealing with novel extraction strategies for bioactive compounds and their applications in the food industry. 5 articles covering the aspects mentioned above were published on this Research Topic after a rigorous single-blind peer-review process. In one of the articles of this Research Topic, Liu L. et al. extracted pectin from *Actinidia arguta Sieb*. et Zucc (*A.arguta*) using the ultrasound-assisted (UA)-acid (UAAE) method and the single acid method. The physicochemical properties, structure, and antioxidant properties of two different pectins were investigated. The results demonstrated that the extraction yield of the UAAE was higher than that of the single acid process. Also, the antioxidant outcomes indicated that the UAE pectin is superior to those of the single acid-extracted pectin. Overall, this study demonstrated that extracted pectin could be used as a natural antioxidant and UAEE is a promising methodology for the extraction of other food additives. Similarly, Mohammed et al. used ultrasonication for 10, 20, and 30 min with ethanol to extract date seed phenolic (DSP) compounds. These authors incorporated dehydrated liposomes (1–3% w/w) into soft cheeses and evaluated their impact on benign prostatic hyperplasia (BPH) using male Sprague-Dawley rats. After inducing BPH, rats were fed these bioactive cheeses for 8 weeks. The findings exhibited that UAE effectively lowered the extraction time of DSP compunds. A high yield of phenolic compounds (558 mg gallic acid/g) and flavonoids (55 mg quercetin/g) with high antioxidant activity (74%) were obtained after 30 min extraction and 50% of ethanol. The study concluded that liposomal encapsulation was a feasible approach for administering DSP in soft cheese.

Another study by Abd-Alrahman et al. investigated the production of natural flavor compounds through the utilization of *Bacillus subtilis*-fermented soybean meal extract and evaluated their biological potential. L-Lysine and L-Threonine were used to fortify soybean meal extract to improve the fermentation process. Two different strains of *Bacillus subtilis* (i.e., NRCH123 and NRCZ144) were evaluated and the volatile profile of fermented soybean meal was analyzed by gas chromatography-mass spectrometry. The fermented soybean extract with *Bacillus subtilis* NRCZ144 fortified with a combination of 2.5% L-Lysine and 2.5% L-threonine (w/w) showed an interesting profile of flavor compounds, with eucalyptol being identified as the most predominant. Finally, the soybean extract was tested against the human liver cancer cell line HepG2, exhibiting anticancer properties, with an IC_50_ value of 2.26 μg/mL. This extract also displayed potent cytotoxic capacity, with an IC_50_ value of 1.02 μg/mL.

Another study by Ling et al. examined the impact of *Flammulina velutipes* polysaccharides (FVPs) on myofibrillar protein (MP) oxidation and physicochemical properties of catfish surimi. FVP was added to surimi at concentrations ranging from 1 to 2%. The degree of MP oxidation and the physicochemical attributes of the surimi were studied, while the microstructure was observed by scanning electron microscopy (SEM). The outcomes revealed that the carbonyl content and the thiobarbituric acid reactive substances (TBARS) in the FVP groups were lower than those observed in the control surimi. Furthermore, the addition of FVP significantly improved the water-holding capacity (WHC), gel strength, elastic modulus (G'), and loss modulus (G“) of surimi, and provided to the surimi gel a stronger continuity and a denser structure. Therefore, FVP would not only exert a cryoprotective impact on surimi, but also improve the quality of surimi, reducing MP oxidation, and decreasing lipid and water loss during frozen storage.

In another study, four methods, namely Standard Normal Variate (SNV), Multiplicative Scatter Correction (MSC), Normalization (Nor), and Savitzky–Golay Smoothing (SG), were evaluated to preprocess near-infrared spectroscopy spectra and determine galactooligosaccharides (GOS), fructooligosaccharides (FOS), calcium (Ca), and vitamin C (Vc) in infant formula milk powders (Liu S. et al.). Additionally, Partial Least Squares Regression (PLSR) and Support Vector Regression (SVR) models were developed to predict the contents of GOS, FOS, Ca, and Vc in the milk powders. The findings demonstrated that after SNV preprocessing, the original spectra of GOS and FOS could effectively extract feature wavelengths using the CARS algorithm, leading to favorable predictive results through the CARS-SVR model. This research offers information that can be used for online nutritional component detection and optimization control in the production process of infant formula.

Overall, the studies listed above provide a variety of approaches for the extraction of bioactive compounds using novel extraction techniques that support sustainability and innovation in the food sector. We are convinced that these articles are an important source of knowledge about how to encourage eco-friendly practices and improve the quality of products.

## Author contributions

AZ: Conceptualization, Writing – original draft. PG-C: Writing – review & editing. BH-L: Writing – review & editing.

## References

[1] Zaky AA Akram MU Rybak K Witrowa-Rajchert D Nowacka M. Bioactive compounds from plants and by-products: Novel extraction methods, applications, and limitations. AIMS Mol Sci. (2024) 11:150–88. 10.3934/molsci.202401036613382

[2] Zaky AA Simal-Gandara J Eun JB Shim JH Abd El-Aty AM. Bioactivities, applications, safety, and health benefits of bioactive peptides from food and by-products: a review. Front Nutr. (2022) 8:815640. 10.3389/fnut.2021.815640 35127796 PMC8810531

